# Outcomes of Nonoperative Management of Penetrating Abdominal Trauma Injury: A Retrospective Study

**DOI:** 10.7759/cureus.58599

**Published:** 2024-04-19

**Authors:** Yasser A Obadiel, Ali Albrashi, Noman Allahabi, Mutahhar Sharafaddeen, Faisal Ahmed

**Affiliations:** 1 General Surgery, Al-Thawra Modern General Hospital, Sana'a University, Sana'a, YEM; 2 Surgery, Faculty of Medicine, 21 September University, Sana'a, YEM; 3 Surgery, General Military Hospital, Sana'a, YEM; 4 Urology, Ibb University, Ibb, YEM

**Keywords:** computed tomography scan, solid abdominal organ injuries, nonoperative management, wounds and injuries, abdominal gunshot wounds, penetrating abdominal injuries, conservative management

## Abstract

Background: The treatment of penetrating abdominal injuries has changed in recent years with more focus on "nonoperative management" (NOM) to avoid unnecessary laparotomies while identifying injuries early. Although the NOM approach is widely used for stab wounds, its effectiveness in managing abdominal gunshot wounds is controversial. NOM of penetrating abdominal injuries is becoming more dependent on hemodynamic stability and improved noninvasive radiological interventions. The role of NOM is significantly underreported and underestimated in developing countries, particularly in fragile and conflict-affected states such as Yemen. The present study aims to evaluate the clinical outcomes of NOM in penetrating abdominal trauma injury patients and identify factors associated with NOM failure in a low-resource setting.

Methods: A retrospective study from January 2021 to December 2022 including patients diagnosed with penetrating abdominal trauma at the General Military Hospital, Sana'a, Yemen, was conducted. Hemodynamically stable patients without peritonitis or clear indications for immediate laparotomy were candidates for NOM and were included in the study. Patients with blunt abdominal injuries, penetrating wounds outside the abdomen, particularly head injury, eviscerated structures, and gastrointestinal hemorrhage, or those pronounced dead on arrival were excluded. The primary outcome was the success and failure rate of NOM necessitating laparotomy. The secondary outcome was the factors associated with NOM failure.

Results: During the study, 256 patients with penetrating abdominal injury were admitted, with 222 (86.7%) undergoing immediate laparotomy and 34 (13.3%) treated with NOM. The mean age was 27.6±7.4 years. Bump explosions, mostly sharp objects (secondary blast injuries), were the main causes of injury (n=18, 52.9%). Other causes were low-velocity gunshot wounds, stab wound injuries, and shotgun injuries in 14 (41.2%), one (2.9%), and one (2.9%), respectively. The majority of patients (n=25, 55.9%) were admitted within 6-24 hours of the incident. The abdominal computed tomography (CT) scan revealed various injuries in all patients, including hemoperitoneum in 11 (32.4%), pneumoperitoneum in five (14.7%), liver injury in 15 (44.1%), foreign body attached to the wall colon in 23 (67.6%), kidney injury in two (5.9%), and splenic injury in one (2.9%). NOM was successful in 31 (91.2%) patients. NOM failed in three (8.8%). One patient was treated via the laparoscopic procedure, and two patients were treated with laparotomy procedures. Five (14.7%) cases required intensive care unit (ICU) admission, with no deaths or major complications. In univariate analysis, the presence of free intra-abdominal fluid (pneumoperitoneum) on the initial CT scan and the need for ICU admission were associated with NOM failure and were statistically significant (p<0.05).

Conclusion: Our findings support that some penetrating abdominal trauma patients can benefit from NOM. The goal of preventing unnecessary laparotomies should be aligned with a comprehensive comprehension of the clinical signs and symptoms of NOM failure and the necessity for surgical intervention. Serial abdominal examinations remain the foundation of selected NOM; nevertheless, radiological and laboratory tests can be important tools in decision-making. In this study, free intra-abdominal fluid on the initial CT scan and the need for ICU admission were associated with NOM failure.

## Introduction

The treatment of individuals with penetrating abdominal injuries has changed in recent years from routine laparotomy to nonoperative management (NOM) in selected cases, thanks to a better understanding of trajectories, potential for organ injury, and correlation with advanced radiographic imaging [1]. In the last decade, "nonoperative management" (NOM) for penetrating abdominal injuries caused by sharp objects has been a widely practiced contemporary approach [1]. Recently, this approach has also been adopted for specific patients who have sustained gunshot wounds to the abdominal region [1,2]. "Nonoperative management" entails a comprehensive evaluation of the trauma to identify any contraindications, the use of computed tomography (CT) scans to assess any internal abdominal injuries, meticulous monitoring of the patient's hemodynamic status, regular physical examinations, and repeated laboratory tests [3]. Contraindications for NOM include unstable hemodynamic conditions, the presence of peritonitis during clinical examination, and simultaneous head injuries or other conditions that hinder accurate and consecutive assessments [4].

A recent systematic review and meta-analysis of outcomes of selective NOM of civilian abdominal gunshot wounds showed that NOMs of abdominal gunshot wounds are safe when conducted in hemodynamically stable patients without a reduced level of consciousness or signs of peritonitis [3]. In the study by Bennett et al. [5], among 62 cases treated with NOM, NOM failed in 14.5%, eventually necessitating laparotomy. Factors associated with NOM failure in this study was the presence of free peritoneal fluid on the initial CT images. Given the relatively high prevalence of penetrating trauma cases in Yemen trauma centers [6], the role of NOM in the Yemeni context has not been thoroughly investigated. Hence, the present study aimed to evaluate the clinical outcomes of NOM in hemodynamically stable patients with penetrating abdominal trauma injury patients and identify factors associated with NOM failure leading to laparotomy in a low-resource setting.

## Materials and methods

Study design and exclusion criteria

A retrospective study between January 2021 and December 2022 including adult patients diagnosed with penetrating abdominal trauma at the General Military Hospital in Sana'a, Yemen, was conducted. Patients with evidence of thoracoabdominal, anterior abdominal, flank, and/or back penetrating injury and were hemodynamically stable without peritonitis, evisceration, or impalement were candidates for NOM and were included in the study. Patients with blunt abdominal injuries, penetrating wounds outside the abdomen, diffuse peritonitis, eviscerated structures, hemodynamic instability, and gastrointestinal hemorrhage, or those pronounced dead on arrival were excluded.

Management protocol

All patients received a comprehensive clinical examination, beginning with ABCDE: (A) airway management and cervical spine protection, (B) breathing and ventilation, (C) circulation with hemorrhage control, (D) disability (assessment of neurological status), and (E) exposure (complete visualization)/environmental control (prevention of hypothermia), which were used to determine hemodynamic stability and awareness level. The general assessment included vital signs (blood pressure, pulse, respiration rate, and state of consciousness) as well as indicators of any accompanying injuries. The local examination included an abdominal check for any signs of related injuries, as well as a local assessment of the wound for penetration. After patient stability, optimal hydration, prophylactic antibiotic administration, and focused assessment with sonography in trauma (FAST) examination, intravenous contrast abdominal computed tomography (CT) scans were performed without oral or rectal contrast. In the absence of CT scan findings suggestive of hollow viscus injury, the patients treated via NOM were admitted to either the intensive care unit (ICU) or the surgical unit, where they received 1:2 nurse care and were monitored continuously. They were treated with serial clinical examinations by the on-call trauma surgeon or senior resident every 2-3 hours, as well as repeat blood tests (white cell counts, hemoglobin, and platelet count) every 6-8 hours.

Study variables

The variables included in this study were sociodemographic variables such as age, sex, and time elapse before presentation to the hospital and clinically related variables such as vital signs before the operation, preoperative hemoglobin, mechanisms of injury, sites of injury and associated injuries, FAST result, CT scan findings, NOM outcome (success or failure), length of hospital stay, ICU admission, and management complications.

Main outcomes

The primary outcome was the success rate and failure rate of NOM necessitating laparotomy (worsening vital signs, signs of peritonitis in clinical examination, or a significant drop of hemoglobin requiring acute blood transfusion of <2 g/dL from baseline) [7]. The secondary outcome was to identify the factors associated with NOM failure.

Statistical analysis

The study used categorical and numerical variables, with frequencies and percentages for categorical variables and means and standard deviations (SDs) for numerical variables. The Mann-Whitney U test and/or independent samples t-test were used to evaluate the association between quantitative factors and NOM failure. Fisher's exact test and/or chi-square analysis were employed to look at the links between qualitative factors and NOM failure. A p-value of less than 0.05 was defined as a statistically significant p-value. The Statistical Package for the Social Sciences (SPSS) version 22 (IBM SPSS Statistics, Armonk, NY) was used for statistical analysis.

Ethical requirements

The study was approved by the Ethics Research Committee of General Military Hospital (ID: 02-2022) (date: 13-1-2022), in compliance with the ethical standards outlined in the Declaration of Helsinki. Informed consents were obtained from all patients included in the study.

## Results

**Figure 1:**
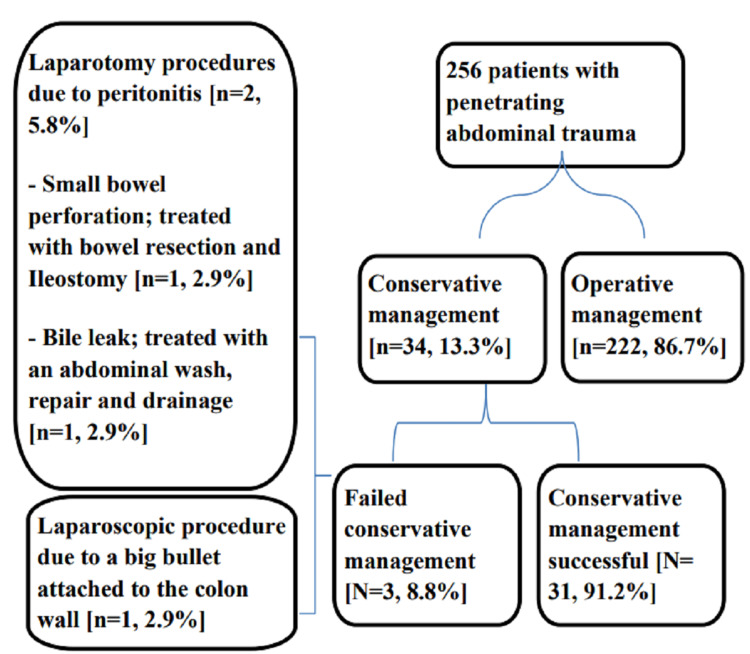
Flowchart of the study patients with penetrating abdominal trauma

A total of 256 patients were admitted with the diagnosis of penetrating abdominal injury during the study period. Of them, 222 (86.7%) underwent immediate laparotomy, the remaining 34 (13.3%) were managed via NOM, and those were included in our study (Figure 1).

The mean age was 27.6±7.4 years (range: 18-45 years). Bump explosions, mostly sharp objects (secondary blast injuries), were the main source of injury in 18 (52.9%) patients. Other causes were low-velocity gunshot wounds, stab wound injuries, and shotgun injuries in 14 (41.2%), one (2.9%), and one (2.9%), respectively. The common location of injury was in the anterior abdominal wall in 11 (32.4%) patients, followed by anterior thoracoabdominal injury in 13 (38.2%) patients. Twenty-five (58.8%) patients presented with associated injuries, including chest injuries in 13 (38.2%) patients, lower extremity injuries in five (14.7%) patients, and upper extremity injuries in two (5.9%) patients. The majority of patients (55.9%) presented between six hours and 24 hours of trauma occurrence.

**Figure 2:**
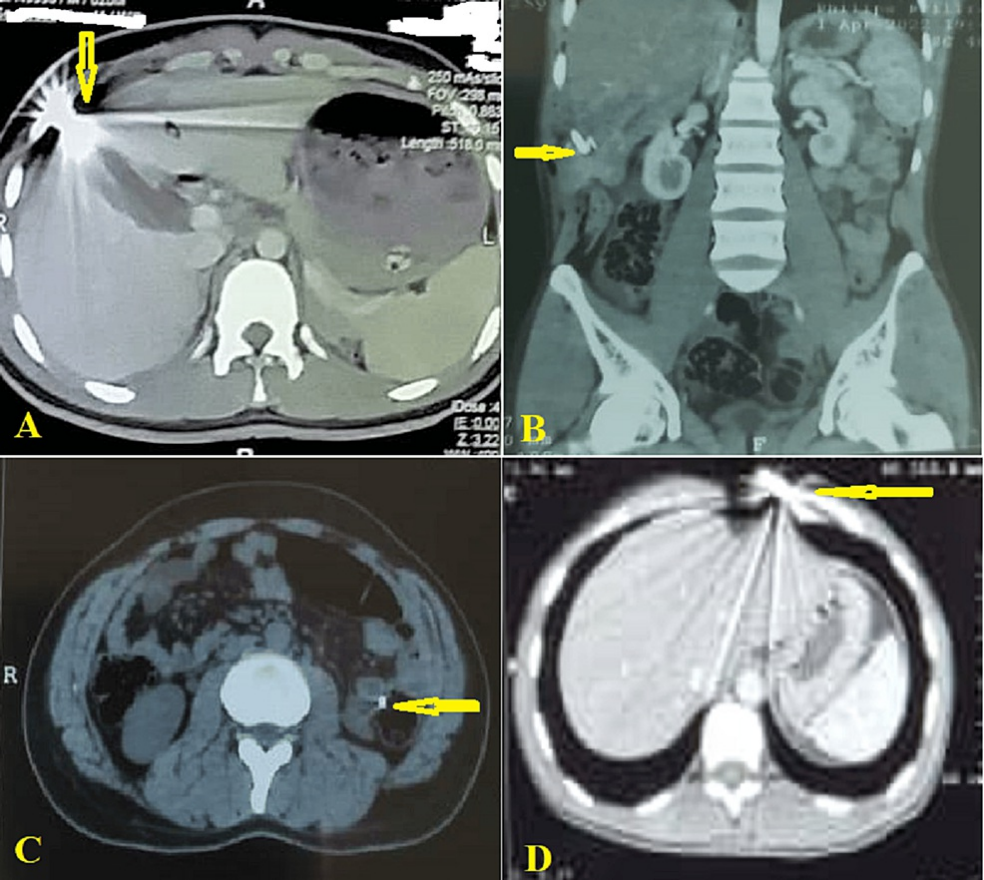
Computed tomography scan showing (A) penetrating gunshot abdominal injury with hemoperitoneum and liver damage (yellow arrow) (axial view), (B) penetrating gunshot abdominal injury with hemoperitoneum and liver damage (yellow arrow) (coronal view), (C) penetrating gunshot abdominal injury with a foreign body in the wall colon (yellow arrow) (axial view), and (D) penetrating gunshot thoracoabdominal injury (yellow arrow) and pneumoperitoneum (axial view)

Regarding physical examination, mild abdominal tenderness was found in 18 (52.9%) patients at the site of injuries, four (11.8%) patients had microscopic hematuria, and three (8.8%) patients needed blood transfusions, two patients when they arrived at the hospital and one patient during hospitalization. FAST examination of the abdomen was performed in all patients. Most of these were positive in 27 (79.4%) patients and were negative in seven (20.6%). The abdominal CT scan revealed various injuries in all patients, including hemoperitoneum in 11 (32.4%), pneumoperitoneum in five (14.7%), liver injury in 15 (44.1%), foreign bodies attached to wall colon in 23 (67.6%), kidney injury in two (5.9%), and splenic injury in one (2.9%) (Figure 2).

Nonoperative management was successful in 31 (91.2%) patients, while it failed in three (8.8%) patients, including one (2.9%) patient who underwent laparoscopy to remove the big bullet from the abdomen and two (5.9%) ended by laparotomy. One (2.9%) patient with peritonitis due to small bowel perforation was treated with bowel resection and ileostomy, and one (2.9%) patient with peritonitis due to bile leak was treated with an abdominal wash and drainage.

Five (14.7%) cases needed ICU admission. The mean hospital stay was 5.3±1.71 days (range: 3-10 days). Surgical site infection was found in one (2.9%) patient, which occurred after five days of laparotomy and was treated with proper antibiotic therapy and serial dressing (Table 1). There were no deaths or major complications.

**Table 1:** Demographic characteristics and presenting features of all patients *All liver and splenic injuries were grade III and IV. **The reported kidney injuries were grade III and IV. CT scan: computed tomography scan, FAST: focused assessment with sonography in trauma, ICU: intensive care units, SD: standard deviation

Variables	Number (%)
Age (year), mean±SD	27.6±7.4
Age group	
Less than 20 years	4 (11.8%)
Between 20 and 30 years	25 (73.5%)
More than 30 years	5 (14.7%)
Gender	
Male	34 (100%)
Number of external injuries	
Single	14 (41.2%)
Multiple	20 (58.8%)
Wound site	
Anterior abdominal area	11 (32.4%)
Flank abdominal area	5 (14.7%)
Back abdominal area	5 (14.7%)
Thoracoabdominal area	13 (38.2%)
Size of wound	
Less than 0.5 cm	2 (5.9%)
Between 0.5 and 1 cm	14 (41.2%)
More than 1 cm	18 (52.9%)
Mechanism of injury	
Stab wound	1 (2.9%)
Gunshot (bullet)	13 (38.2%)
Shotguns	1 (2.9%)
Pump explosion	19 (55.9%)
Associated injury	
Upper extremities	2 (5.9%)
Thoracic "chest tube"	11 (32.4%)
Lower extremities	6 (17.6%)
Multiple injuries	1 (2.9%)
Time from injury to hospital arrival	
Less than 6 hours	2 (5.9%)
Between 6 and 24 hours	19 (55.9%)
More than 24 hours	13 (38.2%)
Symptoms at admission	
Abdominal pain	18 (52.9%)
Hematuria	4 (11.8%)
Need for blood transfusion	3 (8.8%)
FAST ultrasound result	
Positive	27 (79.4%)
Negative	7 (20.6%)
CT findings	
Hemoperitoneum	11 (32.4%)
Pneumoperitoneum	5 (14.7)
Liver injury*	15 (44.1%)
Foreign bodies attached to the wall colon	23 (67.6%)
Splenic injury*	1 (2.9%)
Kidney injury**	2 (5.9%)
ICU admission	5 (14.7%)
Hospital stays	
Between 5 and 7 days	17 (50.0%)
More than 7 days	17 (50.0%)
Complication	1 (2.9%)
Conservative management outcome	
Failure	3 (8.8%)
Successful	31 (91.2%)

In univariate analysis, the presence of free intra-abdominal fluid (pneumoperitoneum) on the initial CT scan and the need for ICU admission were associated with NOM failure and were statistically significant (p<0.05) (Table 2).

**Table 2:** Factors associated with nonoperative management outcome CT scan: computed tomography scan, FAST: focused assessment with sonography in trauma, ICU: intensive care unit, SD: standard deviation Note: Boldface indicates a statistically significant result (p<0.05).

Variables	Subgroups	Conservative management outcome		p-value
		Failure (3 (8.8%))	Success (31 (91.2%))	
Age (year)	Mean (SD)	24.3 (4.7)	27.9 (7.6)	0.432
Age group	<20 years	1 (33.3)	3 (9.7)	0.405
	20-30 years	2 (66.7)	23 (74.2)	
	>30 years	0 (0.0)	5 (16.1)	
Number of external injuries	Single	2 (66.7)	18 (58.1)	1.000
	Multiple	1 (33.3)	13 (41.9)	
Location of injury	Anterior	0 (0.0)	11 (35.5)	0.055
	Flank	2 (66.7)	3 (9.7)	
	Back	0 (0.0)	5 (16.1)	
	Thoracoabdominal	1 (33.3)	12 (38.7)	
Size of exciting wound	<0.5 cm	0 (0.0)	2 (6.5)	0.835
	0.5-1 cm	1 (33.3)	13 (41.9)	
	>1 cm	2 (66.7)	16 (51.6)	
Mechanism of injury	Stab wound	0 (0.0)	1 (3.2)	0.756
	Bullets	2 (66.7)	11 (35.5)	
	Shotguns	0 (0.0)	1 (3.2)	
	Pump explosion	1 (33.3)	18 (58.1)	
Associated injury	Upper extremities	0 (0.0)	2 (6.5)	0.939
	Thoracic	1 (33.3)	10 (32.3)	
	Lower extremities	1 (33.3)	5 (16.1)	
	No	1 (33.3)	13 (41.9)	
	Multiple injuries	0 (0.0)	1 (3.2)	
Time from injury to hospital arrival	Mean (SD)	17.0 (8.7)	19.6 (10.6)	0.682
Abdominal pain	No	2 (66.7)	14 (45.2)	0.915
	Yes	1 (33.3)	17 (54.8)	
Hematuria	No	3 (100.0)	27 (87.1)	1.000
	Yes	0 (0.0)	4 (12.9)	
Need for blood transfusion	No	2 (66.7)	29 (93.5)	0.616
	Yes	1 (33.3)	2 (6.5)	
FAST ultrasound	Positive	3 (100.0)	24 (77.4)	0.860
	Negative	0 (0.0)	7 (22.6)	
Hemoperitoneum in CT scan	No	1 (33.3)	22 (71.0)	0.494
	Yes	2 (66.7)	9 (29.0)	
Pneumoperitoneum in CT scan	Yes	3 (100.0)	2 (6.5)	<0.001
	No	0 (0.0)	29 (93.5)	
Liver injury in CT scan	No	2 (66.7)	17 (54.8)	1.000
	Yes	1 (33.3)	14 (45.2)	
Foreign body attached to the wall colon in CT scan	No	0 (0.0)	11 (35.5)	0.543
	Yes	3 (100.0)	20 (64.5)	
Splenic injury in CT scan	No	3 (100.0)	30 (96.8)	1.000
	Yes	0 (0.0)	1 (3.2)	
Kidney injury in CT scan	No	3 (100.0)	29 (93.5)	1.000
	Yes	0 (0.0)	2 (6.5)	
Hospital stay (days)	<7 days	1 (33.3)	16 (51.6)	1.000
	≥7 days	2 (66.7)	15 (48.4)	
Complication	No	2 (66.7)	31 (100.0)	0.141
	Yes	1 (33.3)	0 (0.0)	
ICU admission	No	0 (0.0)	29 (93.5)	<0.001
	Yes	3 (100.0)	2 (6.5)	

## Discussion

In this study, we retrospectively evaluated the outcome of NOM for penetrating abdominal trauma and identified factors associated with NOM failure in a low-resource setting. The success rate of NOM was 91.2%. Additionally, the presence of free intra-abdominal fluid on the initial CT scan and the need for ICU admission was associated with NOM failure. Nonoperative management has become a widely accepted and well-established technique for treating penetrating abdominal injuries in recent decades [3,8]. It is predicated on the lack of hemodynamic instability and peritonitis, which are relative words that a single value cannot characterize. Age, physiologic conditioning, comorbid disorders, and medications can all affect the determination of NOM, giving deceptive results. Peritonitis is usually defined by each patient's pain threshold, which varies substantially. Astute practitioners must carefully watch patients, examine circumstances holistically, and rely on their knowledge, experience, and surgical abilities to identify whether these conditions exist [9].

In the present study, a total of 256 patients were admitted with the diagnosis of penetrating abdominal injury during the study period. Of them, 222 (86.7%) underwent immediate laparotomy, and the remaining 34 (13.3%) were managed via NOM. As a result, NOM may be particularly useful in situations when medical resources are limited, but the amount of trauma is overwhelming [10]. Our result was in line with previous reports. According to a recent systematic review and meta-analysis, urgent laparotomy remains the primary method for managing penetrating abdominal injuries. Approximately one-third of patients with abdominal gunshot wounds get NOM [10].

In this study, the mean age was 27.6±7.4 years; most patients were aged between 20 and 30 years. All cases were male. Our findings are comparable to the results found by Osinowo et al. [11]. In their study, the reported mean age was 27±10 years, with no specific age group being affected. Additionally, the study showed a male predominance of 88%, which was consistent with our findings. Additionally, in this study, the majority of cases (55.9%) arrived at the hospital after more than six hours but within 24 hours of injury occurrence. Our explanations for this delay are attributed to the time to transfer after the injury as most included cases were soldiers injured in war accidents in conflicted areas. These findings may also justify the admission delay for up to 24 hours in some cases.

In this study, bump explosions, mostly sharp objects (secondary blast injuries), were the main causes of injury (n=18, 52.9%). Other causes were low-velocity gunshot wounds, stab wound injuries, and shotgun injuries in 14 (41.2%), one (2.9%), and one (2.9%), respectively. The findings from this study were predictable as most of the victims were soldiers and were injured during wars. Blast injuries caused the vast majority of injuries to military personnel in recent wars and conflicts [12]. Secondary blast injuries result from debris displaced by the explosion's blast wind, affecting the body surface, either from the explosive device or surrounding material. They are the primary cause of explosion-related injuries, often caused by intentional explosive devices designed to injure as many people as possible, such as nails, metal ball bearings, or screws [12,13].

The most common symptoms in this study were abdominal tenderness. Previous studies reported similar findings [3,5]. The most frequent signs of an intra-abdominal injury are abdominal discomfort, tenderness, guarding, and distention. Other symptoms, such as shortness of breath or chest discomfort, may be connected with serious peritoneal injuries. However, it is important to recall that 40% of individuals with substantial hemoperitoneum show no peritoneal symptoms [3].

Abdominal trauma may be accompanied by various concomitant injuries, complicating the therapy and influencing the prognosis. In the research conducted by Gad et al. [14], skeletal injuries were the most prevalent related injuries, followed by chest injuries. Similarly, 58.8% of our patients experienced various injuries, the majority of which were skeletal, and all were effectively handled

FAST can be a valuable initial diagnostic test following penetrating abdominal injuries. A positive FAST is a high indicator of abdominal damage. However, negative FAST results need to be confirmed with other diagnostic investigations, such as CT scans. Still, due to its low sensitivity, FAST cannot be relied upon for distinguishing penetrating abdominal injury patients who may or may not need surgical exploration. The reported specificity of FAST scanning in penetrating abdominal trauma is 94%-98%, with a sensitivity of just 21%-46% [15,16]. In our study, FAST was negative in seven (20.6%) patients. This may be due to underestimation of the FAST result owing to low experience as most of the FAST was performed by senior surgical residents. A future prospective study may be needed for further evaluation of the role of FAST in selecting patients for NOM. FAST has limited utility in the detection of retroperitoneal hemorrhage and hollow viscus perforations and estimation of intraperitoneal bleeding.

Previous studies focused on CT manifestations of intestinal perforation caused by a variety of factors. However, few previous studies attempted to quantify the most useful findings as free air and fluid. A study by Seishima et al. [17] found that patients with colon perforation had a higher density of ascites. Hainaux et al. [18] found that free air bubbles along the intestine wall and segmental intestinal wall thickening were significant predictors of perforation at the location. Additionally, in a meta-analysis, CT was found to have an accuracy of 94.7% in determining the need for laparotomy in hemodynamically stable patients with penetrating abdominal injury [19]. Pneumoperitoneum, free fluid without solid organ injury, thickened small intestines, and dilated small intestines are common CT findings of hollow viscus injuries [20]. In the present analysis, we found that intra-abdominal free fluid on CT images is associated with NOM failure and the need for laparotomy. Our finding was similar to a previous report [5]. This suggests that clinicians should suspect NOM failure and consider diagnostic laparoscopy or laparotomy [21]. On the other hand, a recent meta-analysis found that the diagnostic accuracy of laparoscopy ranges from 50% to 100%, influenced by surgeon experience, but its reliability in detecting hollow organ injuries is low [22]. The disadvantages of CT scans are limited sensitivity for identifying diaphragmatic, pancreatic, and hollow visceral injuries. Moreover, a CT scan is a costly procedure that needs intravenous and/or oral contrast, which can produce unpleasant responses. In general, radiological imaging should not be blindly trusted, as this can lead to dangerous outcomes [23].

The success rate of NOM in this study was 91.2%. The reported success rate of NOM in penetrating abdominal trauma was similar to previous reports. For example, in the study by Kaur et al. [24], the success rate of NOM was 93.8%. In the study conducted by Navsaria et al. [25], the success rate of NOM was 95.2%. The high success rate in these reports and our study is because of highly selective criteria for NOM in penetrating abdominal injuries. The selection criteria used in the current study proved to be optimal for achieving satisfactory outcomes. It appears rational to promote NOM, particularly in high-volume institutions such as ours, with a high level of competence and adequate surgical facilities. However, our study's results are based on high-volume, level 1 academic trauma facilities. For that, the current findings may not be generalizable, and caution in using NOM for abdominal penetrating wounds should be performed especially in resource-limited settings and in settings lacking computed tomography scans [26].

Continuous clinical monitoring is crucial for conservatively treated patients, as 8.8% required laparotomy in this study. Literature shows similar NOM failure rates [3]. In a comprehensive research by Inaba et al. [27] that included 787 patients with gunshot wounds, 636 individuals were conservatively handled, and the failure rate was 4.6%. The reported failure rates in the studies by DuBose et al. [28] and Habashi et al. [29] and a recent systematic review [3] were 3.5%, 5.7%, and 8.8%, respectively. In a recent systematic review of NOM of abdominal gunshot wounds including 53 studies involving 60,291 participants, the pooled proportions of elective NOM and elective NOM failure were 27% (95% confidence interval (CI): 24%-30%) and 10% (95% CI: 7%-13%), respectively [10]. In contrast, the failure rate of NOM in the study by Bennett et al. [5] was higher than our report (14.5%). This variation in reported NOM failure rate may be due to the small number of patients included in different studies, the surgeon's preference to operate with a small suspension on hollow viscus injuries, and limitations due to institutional underreporting of diagnostic procedures in this dataset. An additional prospective study may be needed to further evaluate the role of NOM in selecting patients with penetrating abdominal trauma.

The length of hospital stay, need for blood transfusion, need for mechanical ventilation, stoma rates, financial burden, and delay in return to work are some morbidities of delayed laparotomy after failed NOM [3]. The most common reasons for NOM failure were continuous or recurrent bleeding and missed bowel injuries [3]. The possibility of missed hollow viscus injuries is one of the challenges of NOM [24]. Patients with hollow viscus injuries and no clinical signs of peritonitis on admission may have small and walled-off perforations, and delay of treatment by a few hours does not result in increased morbidity [7]. In a systematic review, O'Malley et al. [22] reported 83 missed injuries in a cohort of 2,569 patients undergoing diagnostic laparoscopy for penetrating abdominal trauma. In this study, for those with NOM failure, one (2.9%) patient underwent laparoscopy to remove the big bullet from the abdomen, and two (5.9%) ended by laparotomy. One (2.9%) patient with peritonitis due to small bowel perforation was treated with bowel resection and ileostomy, and one (2.9%) patient with peritonitis due to bile leak was treated with an abdominal wash and drainage.

In this study, the need for ICU admission was associated with NOM failure. Our result was similar to the previous report by Hernandez et al. [30]. In this study, the need for ICU admission was associated with operative management needs in univariate analysis, but not mortality.

In a recent systematic review, the reported mean hospital length of stay among patients who underwent NOM was six days versus 10 days if they failed NOM or developed an in-hospital complication [3]. The rate of length of hospital stays and complications in our study was within the range reported in the literature and was not associated with NOM failure [7,24]. Demetriades et al. [7] mentioned a significantly shorter hospital stay in isolated liver injury patients treated via NOM than in patients treated operatively.

Study limitations

The main limitation of the current study was the small number of cases treated with NOM. However, we believe that the current results may inspire modification of the local protocol of management to endorse NOM of penetrating abdominal injuries under precise selective criteria. Additionally, the monocentric and retrospective design of the study renders it vulnerable to selection and misclassification biases. Furthermore, our result lacks robust statistical analysis and other important factors such as comorbidities and laboratory data, which may affect NOM decision. For that, our result needs to be validated in a large cohort study with strict criteria for NOM, including a multicenter setting with different levels of facilities.

## Conclusions

Our findings support that penetrating abdominal trauma patients in the absence of hemodynamic instability, peritonism, organ evisceration, positive radiological findings, or an unreliable clinical examination can benefit from NOM. The goal of preventing unnecessary laparotomies should be aligned with a comprehensive comprehension of the clinical signs and symptoms of NOM failure and the necessity for surgical intervention. Serial abdominal examinations remain the foundation of selected NOM; nevertheless, radiological and laboratory tests can be important tools in decision-making. In this study, free intra-abdominal fluid on the initial CT scan and the need for ICU admission were associated with NOM failure.
